# Mercury methylation by metabolically versatile and cosmopolitan marine bacteria

**DOI:** 10.1038/s41396-020-00889-4

**Published:** 2021-01-27

**Authors:** Heyu Lin, David B. Ascher, Yoochan Myung, Carl H. Lamborg, Steven J. Hallam, Caitlin M. Gionfriddo, Kathryn E. Holt, John W. Moreau

**Affiliations:** 1 School of Earth Sciences, The University of Melbourne, Parkville, VIC 3010 Australia; 2 Structural Biology and Bioinformatics, Department of Biochemistry and Molecular Biology, Bio21 Molecular Science and Biotechnology Institute, The University of Melbourne, Parkville, VIC 3010 Australia; 3 Computational Biology and Clinical Informatics, Baker Heart and Diabetes Institute, PO Box 6492, Melbourne, VIC 3004 Australia; 4 Department of Ocean Sciences, University of California, Santa Cruz, CA 95064 USA; 5 Department of Microbiology and Immunology, University of British Columbia, Vancouver, BC V6T 1Z1 Canada; 6 Genome Science and Technology Program, University of British Columbia, Vancouver, BC V6T 1Z4 Canada; 7 Biosciences Division, Oak Ridge National Laboratory, PO Box 2008, Oak Ridge, TN 37831 USA; 8 Department of Infectious Diseases, Central Clinical School, Monash University, Monash, VIC 3800 Australia; 9 Department of Infection Biology, London School of Hygiene & Tropical Medicine, London, WC1E 7HT UK; 10 Present Address: Smithsonian Environmental Research Center, Edgewater, MD 21037 USA; 11 Present Address: Currently at School of Geographical & Earth Sciences, University of Glasgow, Glasgow, G12 8QQ UK

**Keywords:** Microbial ecology, Water microbiology, Structural biology, Biogeochemistry

## Abstract

Microbes transform aqueous mercury (Hg) into methylmercury (MeHg), a potent neurotoxin that accumulates in terrestrial and marine food webs, with potential impacts on human health. This process requires the gene pair *hgcAB*, which encodes for proteins that actuate Hg methylation, and has been well described for anoxic environments. However, recent studies report potential MeHg formation in suboxic seawater, although the microorganisms involved remain poorly understood. In this study, we conducted large-scale multi-omic analyses to search for putative microbial Hg methylators along defined redox gradients in Saanich Inlet, British Columbia, a model natural ecosystem with previously measured Hg and MeHg concentration profiles. Analysis of gene expression profiles along the redoxcline identified several putative Hg methylating microbial groups, including Calditrichaeota, SAR324 and Marinimicrobia, with the last the most active based on *hgc* transcription levels. Marinimicrobia *hgc* genes were identified from multiple publicly available marine metagenomes, consistent with a potential key role in marine Hg methylation. Computational homology modelling predicts that Marinimicrobia HgcAB proteins contain the highly conserved amino acid sites and folding structures required for functional Hg methylation. Furthermore, a number of terminal oxidases from aerobic respiratory chains were associated with several putative novel Hg methylators. Our findings thus reveal potential novel marine Hg-methylating microorganisms with a greater oxygen tolerance and broader habitat range than previously recognized.

## Introduction

Mercury (Hg), a highly toxic metal, is widespread in the environment from primarily anthropogenic sources, leading to increased public concern over the past few decades. [e.g., 1–3]. Methylmercury (MeHg) is recognized as a potent neurotoxin that bioaccumulates through both marine and terrestrial food webs [2, 4], with potential deleterious impacts on human health. With implementation of the Minamata Convention on Mercury [3], a better understanding is expected of potential MeHg sources, in the context of global biogeochemical cycles and factors influencing Hg speciation. For example, oxygen gradients in seawater have been observed to expand to both a wider depth range and larger geographical area in response to climate change [5], and the resulting impacts on biogeochemical cycles, e.g., carbon, nitrogen, and sulfur, have been studied [6]. Effects on Hg cycling, however, are rarely considered.

The environmental transformation of Hg(II) to MeHg is a microbially-mediated process, for which the proteins are encoded by the two-gene cluster *hgcA* and *hgcB* [7]. Possession of the *hgcAB* gene pair is a predictor for Hg methylation capability [8], and the discovery of *hgc* genes has stimulated a search for potential Hg methylating microbes in diverse environments [9]. To date, all experimentally confirmed Hg methylators are anaerobes [10] from three *Deltaproteobacteria* clades (sulfate-reducing bacteria, Fe-reducing bacteria and syntrophic bacteria), one clade belonging to fermentative Firmicutes, and an archaeal clade (*Methanomicrobia*). These microorganisms are ubiquitous in soils, sediments, seawater, freshwater, and extreme environments, as well as the digestive tracts of some animals [9]. A few other *hgc* carriers, not only from anaerobic but also microaerobic habitats, were discovered using culture-independent approaches, including Chloroflexi, Chrysiogenetes, Spirochaetes [9]*, Nitrospina* [11, 12], and Verrucomicrobia [13]. Two more recent studies have expanded the Hg-methylating community [14, 15] and revealed the role played by Hg methylators in the context of global biogeochemical cycling.

Apart from Hg methylation, MeHg demethylation also plays a key role in global Hg cycling. This process is mainly catalyzed by alkylmercury lyase encoded by the *merB* gene, which has been largely found in aerobes (e.g., *Bacillus cereus*, *Staphylococcus aureus*, and *Escherichia coli*). Yet some obligate and facultative anaerobes (e.g., *Geobacter bemidjiensis* and *Geobacter sulfurreducens*) also possess *merB* and can contribute to demethylation [16] and therefore total environmental MeHg levels.

A global Hg survey [17] found a prevalence for MeHg in suboxic waters, where dissolved O_2_ and H_2_S concentrations are typically very low (<50 μm and <10 μm, respectively), especially in regionally widespread oxygen gradients at upper and intermediate ocean depths. Oxygen concentrations can be reduced by a combination of physical and biological forcing effects [6, 18]. These gradients can exist as permanent features in the water column, impinge on coastal margins, or manifest more transiently (e.g., induced by phytoplankton blooms). As oxygen levels decrease, metabolic energy gets increasingly diverted to alternative electron acceptors, resulting in coupling of other biogeochemical cycles, e.g., C, N, S, Fe, and Mn [6, 19, 20]. Recent findings of microaerophilic microbial Hg methylation potential in sea ice and seawater [e.g., 11, 21–23] raise the possibility that this process contributes significantly to ocean MeHg biomagnification.

In this study, we measured the concentrations of Hg (total) and MeHg in the waters of Saanich Inlet, a seasonally anoxic fjord on the coast of Vancouver Island (British Columbia, Canada), and performed targeted metagenomic and metatranscriptomic analyses of seawater sampled from varying depths. Saanich Inlet, as a model natural ecosystem for studying microbial activity along defined oxygen gradients [24], provides an ideal site to search for novel putative Hg methylators and study microbially-mediated Hg cycling in low-oxygen environments. Physico-chemical and biogeochemical parameters, including nutrient and dissolved gas concentrations, have been published from data acquired from 2006 to 2014 [25]. This study clearly illustrates the dynamic character of the oxygen gradient in Saanich Inlet waters. Computational homology modelling was performed to predict the functionality of HgcAB proteins encoded for by putative novel Hg-methylators. Finally, we scanned global metagenomic datasets for recognizable *hgcA* genes, to reassess the environmental distribution of microbial mercury methylation potential.

## Results and discussion

### Hg and MeHg concentrations along redox gradients in Saanich Inlet

**Fig. 1:**
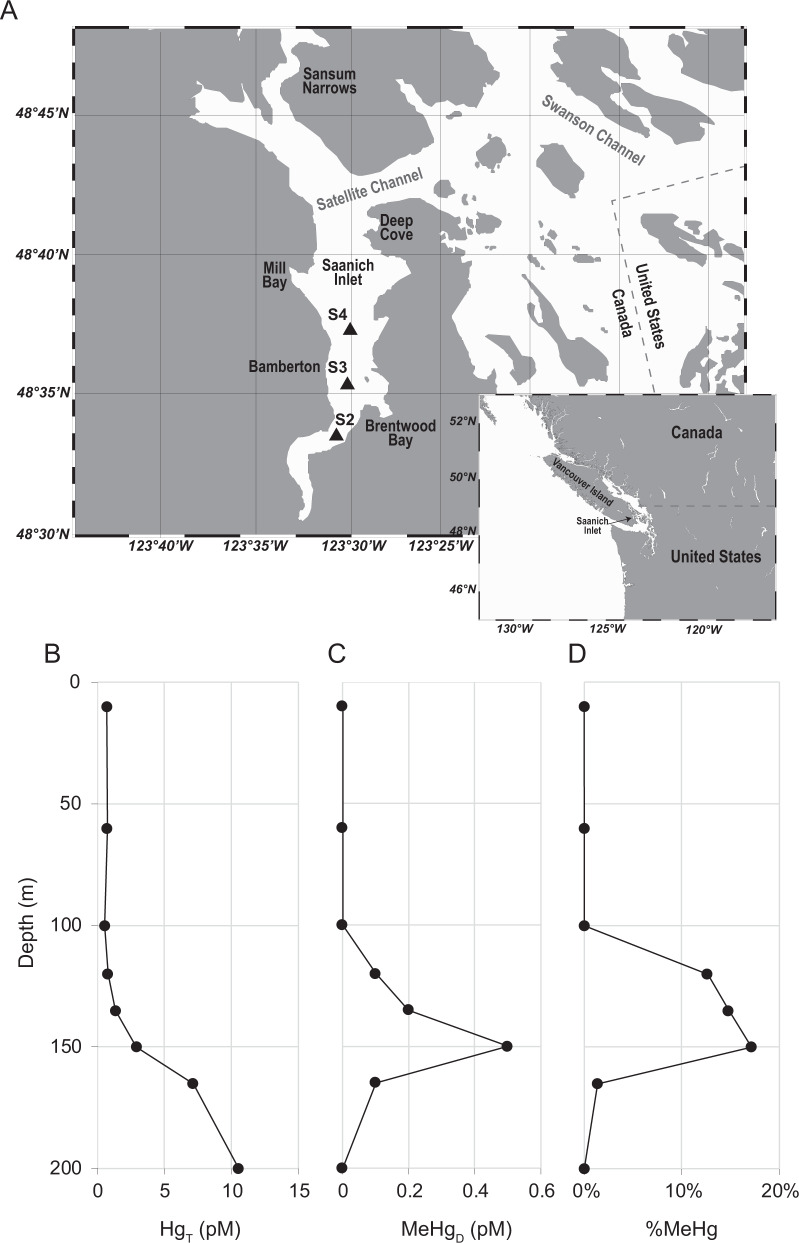
Map of Saanich Inlet and mercury profiles of station “S3”. **A** Map of Saanich Inlet showing locations of the station “S2”, “S3” and “S4” by triangles. **B** Concentration of total dissolved Hg at station “S3”. **C** Concentration of dissolved MeHg at station “S3”. **D** MeHg as a percentage of total dissolved Hg at station “S3”.

Concentrations of total dissolved Hg (Hg_T_) and monomethylmercury (MeHg) from filtered Saanich Inlet S3 station (see Fig. 1A for a map) seawater samples, obtained and analyzed in March–April of 2010, were vertically profiled at eight different depths from surface (10 m) to bottom (200 m) waters. The concentration of Hg_T_ at sea surface was ~0.70 pM and remained nearly constant in seawater above 120 m depth, increasing to 1.35 pM and ~10.56 pM at 135 m and 200 m depths, respectively (Fig. 1B). MeHg was below detection limit (<0.1 pM) for seawater above 100 m depth, but increased to 0.50 pM (17.2% of total Hg) at 150 m depth. However, MeHg then decreased to 0.1 pM at 165 m depth, becoming undetectable at 200 m (Fig. 1C and 1D). Similar MeHg peaks under suboxic conditions have been observed in other seawater depth profiles: the Pacific Ocean [26], Arctic Ocean [27], Southern Ocean [28], Arabian Sea [29], and Mediterranean Sea [30].

### MAG (metagenome-assembled genome) reconstruction and putative Hg methylator identification

A total of 2088 MAGs with completeness >70% and contamination <5% were recovered from all Saanich Inlet metagenomic datasets (generated across all sampling depths, 96% being from station S3, obtained from 2008 to 2013; [24]). From these, 56 MAGs, belonging to seven phyla: Proteobacteria, Marinimicrobia, Verrucomicrobia, Firmicutes, Calditrichaeota, Spirochaetes, and Nitrospinae (Table S1), were identified as having genes homologous with *hgcA* (Fig. S1). Among these, Marinimicrobia and Calditrichaeota have not previously been implicated in Hg methylation. No *hgcAB* genes were recovered from archaeal MAGs, suggesting that archaeal Hg methylators were rare or absent. As most known Hg-methylating microorganisms derive from a limited number of phylogenetic groups [8, 14, 15], our findings expand the database of putative microbial Hg-methylators and their microenvironments.

**Fig. 2:**
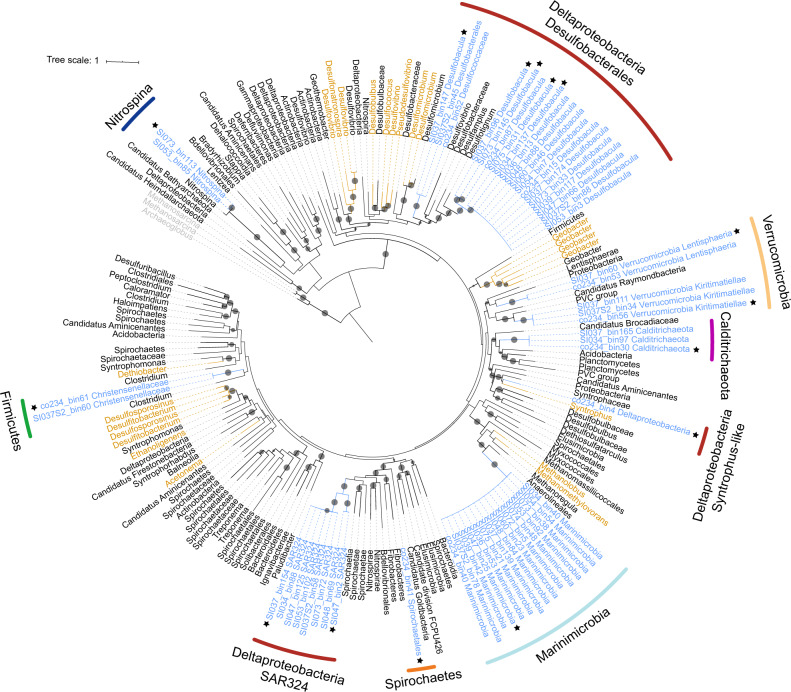
Maximum-likelihood phylogenetic tree of HgcA amino acid sequences (1000 ultrafast bootstrap replicates; values >90% are shown by black dots at the nodes). HgcA sequences recovered in this study are highlighted in blue. HgcA sequences retrieved from public databases are shown in black. Experimentally confirmed HgcA from previous studies are shown in brown. HgcA paralogues from non-methylating bacteria were used as outgroups and are shown in gray. The 15 representative HgcA sequences used in this study are indicated by stars. Taxonomic classifications of the *hgcA*-carrying genomes are labeled in the outer circle by different colors. Scale bar indicates substitutions per site.

Fifteen *hgcA*-carrying MAGs represented members of Marinimicrobia, a widespread but uncultured marine phylum that couples C, N, and S cycling [31]. These Marinimicrobia contained the same *hgcA* gene sequence (100% nucleotide identity), and uniformly possessed *hgcB* genes downstream of *hgcA*. Marinimicrobia genome association with *hgcAB* was supported strongly by emergent self-organizing maps (ESOMs; Fig. S2A). One Marinimicrobia*-*associated MAG, SI037_bin139, exhibited the highest binning quality score (Table S1), with 97.8% completeness and 0% contamination, as estimated by CheckM, having 75 contigs in total. Many Marinimicrobia MAGs also carried 16S rRNA genes, further supporting taxonomic classification (Fig. S3). Notably, Marinimicrobia-HgcA sequences fell within the *Euryarchaeota* in the HgcA phylogenetic tree (Fig. 2; see Fig. S4 for a more detailed tree with accession numbers and strain names), suggesting a different evolutionary pathway to other bacterial *hgc* sequences.

Three MAGs containing *hgcAB* genes were associated with Calditrichaeota, another cosmopolitan marine phylum [32]. HgcA sequences from Calditrichaeota formed a cluster with those of the PVC group, Planctomycetes, Acidobacteria, and Candidatus *Brocadiaceae*, which also have close phylogenetic relationships to *Deltaproteobacteria*, as shown in Fig. 2. ESOMs were constructed (Fig. S2B) that supported the presence of *hgcAB* genes in Calditrichaeota MAGs.

HgcA sequences were found in eight Proteobacteria MAGs, all belonging to the *Deltaproteobacteria* group SAR324. *Deltaproteobacteria* are the most diverse Hg-methylating clade currently known [9]. However, the SAR324 *hgcA* sequences clustered separately from those of three previously described *Deltaproteobacteria* methylating clades (*Desulfovibrio*-like, *Geobacter*-like, and *Syntrophus*-like) [8, 9], forming a potentially novel clade of Hg-methylators (Fig. 2). SAR324 is affiliated with a group of *Deltaproteobacteria* abundant in the deep sea and in low-oxygen settings, with the ability to metabolize sulfur, organic carbon, and C_1_ compounds [33]. A contiguous *hgcB* gene was also recognized downstream of *hgcA* in SAR324 MAGs SI047_bin2 and SI048_bin69. Six other SAR324 MAGs did not contain recognizable *hgcB* genes, but several *hgcB*-like candidates encoding for tandem [CX2CX2CX3C] motifs were recognized in different contigs in the genome. The presence of *hgc* genes in SAR324 MAGs was well supported by ESOM (Fig. S2A).

### Relative abundance of *hgcA* genes and transcripts in Saanich Inlet

**Fig. 3:**
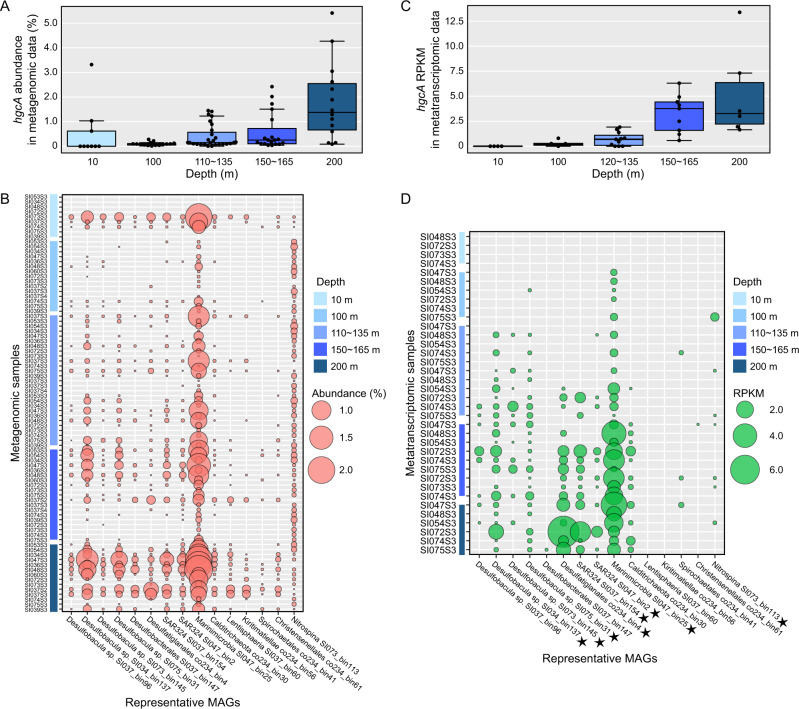
Relative abundance of representative *hgcA* genes and transcripts in different samples from Saanich Inlet. Gene abundance is normalized by gene length and genome equivalent for each sample, and transcript abundances are represented as RPKM values. **A** Relative abundance of the sum of representative *hgcA* genes for different depths. **B** Relative abundance of different representative *hgcA* genes from different metagenomic datasets. Larger circles indicate a higher percentage of the whole microbial community. Different shades of blue indicate different depths from which the samples were taken. **C** RPKM values of the sum of representative *hgcA* transcripts in different depths. **D** RPKM values of different representative *hgcA* transcripts in different metatranscriptomic samples. HgcA sequences from metaproteomic samples are indicated with stars.

The 56 *hgcA* sequences were clustered into 15 groups using a 99% sequence identity threshold. One *hgcA* sequence was selected arbitrarily from each group, with corresponding MAG (Table 1; also indicated by stars in Fig. 2), for further analysis. These sequences were used to recruit reads from Saanich Inlet metagenomic and metatranscriptomic datasets, in order to calculate relative abundance and expression. Results showed that *hgcA* genes were widely distributed in Saanich Inlet water samples, and the abundance of *hgcA* gene copies increased with depth, peaking at ~200 m depth (Fig. 3A). Since *hgcA* is usually present as a single copy per genome, this finding suggests that Hg methylators were most abundant at this depth. Notably, Marinimicrobia was the most abundant putative Hg-methylator detected throughout the water column, accounting for >2% of the microbial community for many samples from 200 m depth (Fig. 3B). *Deltaproteobacteria* with *hgcA* also showed higher abundance in 200 m depth samples, suggesting an overlapping habitat range with Marinimicrobia. Although *hgcA*-carrying *Nitrospina* was not a dominant phylum in Saanich Inlet datasets, the relative abundance of *Nitrospina-*hosted *hgcA* remained nearly constant from sea surface to bottom water, implying the adaptability of this phylum across the redoxcline.

**Table 1:** Summary of 15 representative *hgcA*-carrying MAGs^a^.

MAGs	%Compl.^b^	%Conta.^b^	Size (Mbp)	# Contigs	%GC	16S	Taxonomy
SI034_bin137	**97.58**	**2.992**	4.94	304	40		*Desulfobacula sp*.
SI037_bin96	71.02	**3.87**	2.76	633	39.5		*Desulfobacula sp*.
SI073_bin145	87.48	5.322	4.26	657	39.2		*Desulfobacula sp*.
SI075_bin31	88.61	**2.293**	4.89	394	40.1		*Desulfobacula sp*.
co234_bin4	81.15	5.545	5.91	1000	50.1		Order *Desulfatiglandales*
SI037_bin147	89.67	**1.935**	3.28	289	38.6		Order *Desulfobacterales*
SI037_bin154	89.02	**3.361**	7.85	512	45.5		SAR324 group
SI047_bin2	74.15	**2.941**	3.63	142	36.2		SAR324 group
SI047_bin25	**96.7**	**0**	3.17	92	37.4	+	Phylum Marinimicrobia
co234_bin30	**96.08**	**1.648**	4.54	293	42.9		Phylum Calditrichaeota
SI037_bin60	83.67	**3.914**	8.81	2073	63.9		Class *Lentisphaeria*
co234_bin56	**95.6**	**2.027**	4.85	162	60.6	+	Class *Kiritimatiellae*
co234_bin61	**98.25**	**0**	2.67	97	47.7		Order *Christensenellales*
co234_bin41	72.27	**1.253**	4.68	1025	48.9		Order *Spirochaetales*
SI073_bin113	74.89	**1.709**	2.92	321	43.1		*Nitrospina sp*.

^a^Representative MAGs selected by 99% *hgcA* identity threshold. See Table S1 for all 56 *hgcA*-carrying MAGs.

^b^Completeness above 95% and contamination below 5% are shown in bold; quality of MAGs was determined by CheckM with the “lineage_wf” pipeline.

**Fig. 4:**
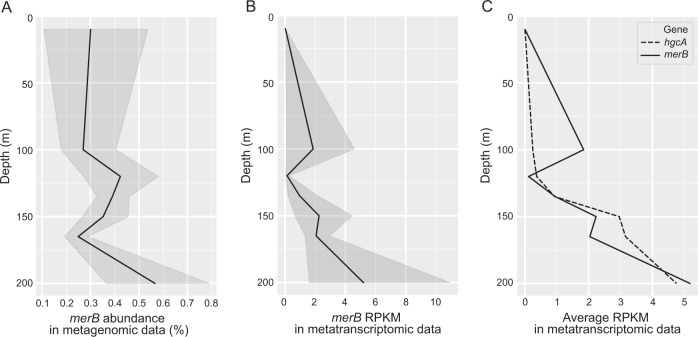
DNA and RNA abundance of *merB* in Saanich Inlet datasets changing with depth. **A** *merB* gene abundance in Saanich Inlet metagenomic datasets. Percentage abundance was normalized by gene length and genome equivalent for each sample. Line plot depicts the average abundance of *merB* with depth; shaded area depicts 95% confidence interval. **B** *merB* transcript abundance in Saanich Inlet metatranscriptomic datasets, as represented by RPKM values. Line plot depicts mean RPKM value of *merB* with depth; shaded area depicts 95% confidence interval. **C** Comparison between RPKM values for *hgcA* (orange) and *merB* (green) in Saanich Inlet metatranscriptomic datasets.

Reads per kilobase per million mapped reads (RPKM) values were calculated for *hgcA* transcripts in each sample to assess *hgc* gene expression in a subset of 36 samples from site S3 (Fig. 3C). Consistent with relative DNA abundance, *hgcA* transcripts also increased with depth to a maximum at 200 m (mean RPKM = 5.1); the most abundant *hgcA* transcripts were associated with Marinimicrobia (Fig. 3D). The abundance of *hgcA* transcript was normalized to housekeeping genes *rpoB* and *gyrB* to study the *hgcA* expression profile (Figure S5). Intriguingly, both the ratio of *hgcA*/*rpoB* RPKM and *hgcA*/*gyrB* RPKM increased with depth, but were similar within uncertainties for bottom waters (from 0 to ~0.6 and ~1.0, respectively). Considering the substantial increase of total *hgcA* transcripts with depth (Fig. 4C) this observation suggests a relatively constant rate of *hgcA* expression by individual microorganisms. Therefore, the increase in *hgcA* transcripts is likely explained by increasing abundance of Hg methylators rather than by an increase in cell-specific *hgcA* expression. This result is consistent with previous studies that showed *hgcA* expression is constitutive [34, 35]. Furthermore, no *hgcA* transcripts were recovered from 10 m depth samples, although a number of *hgc* DNA sequences were recovered. In addition, no *hgcA* transcript associated with Verrucomicrobia (*Lentisphaeria* and *Kiritimatiellae*) was detected for any dataset, which may reflect the relatively lower abundance of Verrucomicrobia*-hgcA* genes, as well as a low transcription level. Predicted amino acid sequences from Saanich Inlet metaproteomic datasets were also scanned for expression of putative *hgcA* genes (Fig. S6; also indicated by stars in Fig. 3D). Excepting from phyla Calditrichaeota, Verrucomicrobia, Spirochaetes, and Firmicutes, representative HgcA sequences could be detected in matched metaproteomic datasets. Notably, of many *hgcA* DNA sequences from the upper depths of Saanich Inlet, only a small number were actively expressed (Fig. 3C and 3D), suggesting an influence of oxygen level or other environmental factors. We therefore caution that abundance of *hgcA* genes, as measured by metagenomic or amplicon sequencing, cannot effectively predict the actual extent of MeHg production.

### Abundance of *merB* genes and transcripts in Saanich Inlet

As MeHg concentrations in the Saanich Inlet water column may reflect net Hg methylation and demethylation, the distribution with depth of *merB*, a gene encoding for an organomercury lyase, was also assessed. Results showed the relative abundance of *merB* genes peaked at 200 m depth, as ~0.6% of total microbial community genes (Fig. 4A). RPKM values of *merB* transcripts in each metatranscriptomic sample were calculated and compared with *hgcA* transcripts. Consistent with metagenomic results, *merB* transcripts peaked at 200 m depth (mean RPKM = 5.2), whereas no *merB* transcripts were detected at 10 m depth (Fig. 4B). Average RPKM values of *merB* were higher than those of *hgcA* at depths <120 m, but were exceeded by *hgcA* RPKM values at 120–200 m depth. Interestingly, the average RPKM of *hgcA* was again exceeded by that of *merB* at 200 m depth, although transcription of both genes increased to maxima at that same depth (Fig. 4C). Contrasting the relative abundance of potential methylators observed in the benthic zone, the observed increased abundance of *merB* genes and transcripts may provide a viable hypothesis for why MeHg levels decreased over certain depth ranges (Fig. 1D).

### Phylogenetic analysis of *hgcA*-carrying Marinimicrobia

**Fig. 5:**
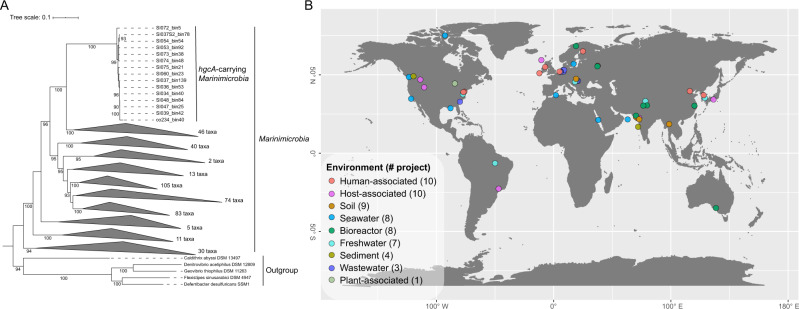
Phylogeny and global distribution of phylum Marinimicrobia. **A** Maximum-likelihood phylogenetic tree of phylum Marinimicrobia based on concatenated housekeeping genes (1000 ultrafast bootstrap replicates; values >90% are shown at the nodes). Scale bar indicates substitutions per site. A total of 424 Marinimicrobia genomes were used for the tree; Marinimicrobia lineages without *hgcA* genes were collapsed to simplify presentation (see Fig. S6 for a more detailed tree). **B** Distribution of *hgcA*-carrying Marinimicrobia in various environments globally, shown as different colors; total numbers of BioProjects from each environment are shown in brackets.

All 15 *hgcA*-carrying Marinimicrobia and 409 non-*hgcA*-carrying Marinimicrobia spp., derived from this study and public databases (NCBI and IMG), were used to build a phylogenetic tree based on concatenated alignment of housekeeping genes (Fig. 5A, Fig. S7). All 15 *hgcA*-carrying Marinimicrobia clustered into a monophyletic clade and carried identical *hgcA* gene sequences, consistent with a single horizontal transfer of *hgcA* into a sublineage of Marinimicrobia. Furthermore, while all Marinimicrobia-associated *hgcA* sequences were identical, four different 16S rRNA genes were represented by these MAGs, with minimum identity of 96.0% (Fig. S3). The four representative 16S sequences from *hgcA*-carrying Marinimicrobia were further searched in the SILVA database [36], and all of them have best hits associated with Marinimicrobia phyla, with identities ranging from 91.17 to 95.71%. This finding suggests that *hgcA*-carrying Marinimicrobia represent a novel candidate species or genus, according to Yarza et al. [37].

### Marinimicrobia is a widespread potential Hg methylator

The phylum Marinimicrobia, a yet uncultivated but apparently cosmopolitan marine group, is thought to play an important role in global biogeochemical cycling, especially across redox gradients [31, 38]. However, the Hg-methylation potential of Marinimicrobia has not previously been recognized. A few HgcA sequences from Saanich Inlet were discovered by Podar et al. [9] from metagenomic analyses; these previously unidentified sequences are shown here to co-locate phylogenetically with the Marinimicrobia-HgcA identified in this study at whole genome resolution. In order to explore the global distribution of *hgcA*-carrying Marinimicrobia, a wider search was performed against NCBI SRA metagenomic datasets. In total, 58 BioProjects from a range of environments, including seawater, soil, sediment, freshwater, industrial wastewater, and plant rhizomes, contained Marinimicrobia*-hgcA* associated reads (Fig. 5B, Table S2), suggesting a wide ecological distribution. Interestingly, our findings of *hgcA*-carrying Marinimicrobia from metagenomic datasets from Gulf of Mexico (PRJNA288120) and Canadian Arctic (PRJNA266338) waters contaminated by oil spills [39, 40] may provide one explanation for observed in situ MeHg production [41].

### Homology models of Marinimicrobia-HgcA and -HgcB proteins

**Fig. 6:**
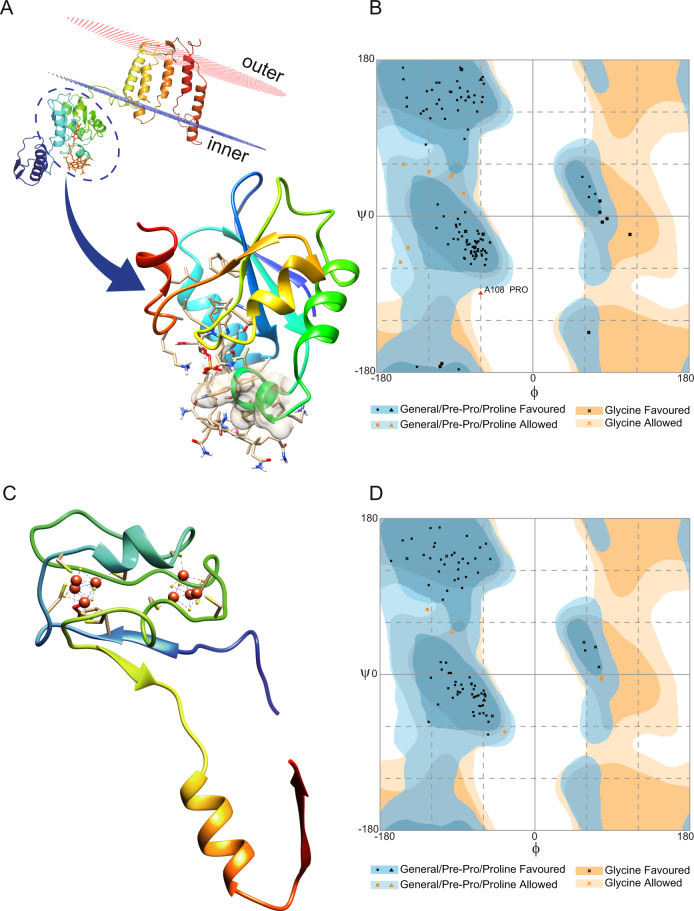
Three-dimensional homology models of Marinimicrobia-HgcA and -HgcB proteins. **A** Model of full-length Marinimicrobia-HgcA, shown relative to the cell membrane. An enlarged view of the functional domain bound to cobalamin is shown, with the ionic interaction between cysteine and cobalt represented by a dotted line. **B** Ramachandran plot of Marinimicrobia-HgcA model showing that 93.2%, 5.9%, and 0.8% of residues lie within favoured, allowed, and outlier regions, respectively. The backbone Phi and Psi (φ and ψ) dihedral angles are shown, along with the energetically favoured regions. **C** Model of full-length Marinimicrobia-HgcB in complex with two [4Fe4S] clusters. Interactions between the protein and iron are shown by dotted lines. **D** Ramachandran plot of Marinimicrobia-HgcB model is shown, revealing 94.1%, 5.9%, and 0% of residues lay within favoured, allowed, and outlier regions, respectively.

As no cultivated representative of Marinimicrobia is currently available to assay for functional Hg methylation, we constructed homology models to test the hypothesis that HgcA and HgcB proteins encoded for by Marinimicrobia possess the three-dimensional structural functionality required for methylating Hg. Analysis of the putative Marinimicrobia-HgcA sequence revealed a comparable domain structure (Fig. 6A) to the previously characterized HgcA from the functionally validated Hg-methylating strain *Desulfovibrio desulfuricans* ND132 [7], including the presence of a globular domain at the N-terminus and five transmembrane spanning helices at the C-terminus. The cap-helix structure and coordinated Cys residues required for Hg(II) methylation by ND132 were conserved in the globular domain of our putative Marinimicrobia-HgcA (Fig. S8A and Fig. S8B), consistent with a conserved catalytic mechanism. The structure of ND132-HgcA was solved bound to the co-factor cobalamin required for catalytic methylation activity. The cobalamin binding pocket for ND132 was comparable to that of the Marinimicrobia-HgcA homology model (726.5 Å^3^ and 678.4 Å^3^, respectively), with 69% conservation of residues interacting with cobalamin. Modelling of cobalamin in the binding site of the Marinimicrobia-HgcA homology model revealed a similar H-bonding network for the two structures (Table S3). Modelling of the Marinimicrobia-HgcB protein (Fig. 6C) demonstrated binding with 2[4Fe-4S] clusters at the N-terminal, similar to the structure of ND132-HgcB (Fig. S8C and Fig. S8D). The conserved two ferredoxin motifs (CX2CX2CX3C), and the C-terminal Cys tail in ND132-HgcB required for potentially transferring electrons and binding the Hg(II) substrate [7, 42], were also observed for Marinimicrobia-HgcB. The structural qualities of Marinimicrobia-HgcA and -HgcB were further compared using Ramachandran plots (Fig. 6B and 6D), with most residues located in the favored or allowed regions in both structures. Overall, this analysis strongly supports the conservation of Hg(II) methylation activity for the putative Marinimicrobia-HgcA and -HgcB proteins. In addition, similar HgcAB homology models constructed for novel putative Hg methylators Calditrichaeota and SAR324 also support functionality (Fig. S9). The same methodology has been adapted to infer functionality for HgcAB proteins from *Nitrospina*, another uncultivated potential Hg methylator [11]. This approach represents a promising method for predicting the functionality of proteins encoded for by uncultivated microorganisms.

### Genome composition and adaptation strategies of *hgcA*-carrying MAGs

**Fig. 7:**
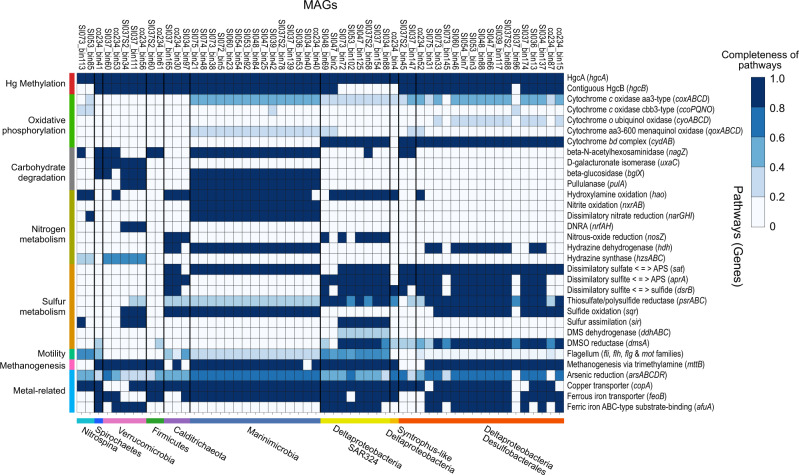
KEGG pathways of the *hgcA*-carrying MAGs. Taxonomic classifications of MAGs are represented at bottom of heatmap by different colors. Categories of pathways are represented at left side of heatmap by different colors. Color of each cell refers to completeness of enzymes involved in each pathway. Corresponding genes involved in each pathway are shown in parentheses.

Metabolic pathways were inferred for MAGs by reference to Kyoto Encyclopedia of Genes and Genomes (KEGG) annotations (Fig. 7). Hg methylators living in oxygen gradients, such as those observed in Saanich Inlet [25], may require the capability to tolerate intermittent lower levels of oxygen exposure. Genes encoding for multiple terminal oxidases were detected in the *hgcA*-carrying MAGs. Specifically, all *hgcA*-carrying Marinimicrobia and most *Deltaproteobacteria* contained at least one gene encoding for cytochrome *c* oxidase *aa3*-type (*coxABCD*), a canonical heme-copper coupling O_2_ reduction to transmembrane proton pumping [43]. Other terminal oxidase systems were also recognized; for example, genes encoding for cytochrome *c cbb*_3_-type (*ccoPQNO*) oxidase were found in several members of *Deltaproteobacteria*, Marinimicrobia, and *Nitrospina*; this oxidase has been shown to exhibit high affinity for oxygen, enabling bacteria to respire O_2_ under both oxic and suboxic conditions [44, 45]. All *hgcA*-carrying SAR324, and the majority of other *hgcA*-carrying *Deltaproteobacteria*, contained the complete operon encoding for cytochrome *bd* complex (*cydAB*); this type of oxidase has also been demonstrated to support adaptation to low-oxygen environments, because of its relatively high affinity for O_2_ [46]. In addition, cytochrome *o* oxidase (*cyoABCD*), carried by many *Deltaproteobacteria*, is an enzyme proposed to function under O_2_-rich conditions [47]. All Marinimicrobia and some *Deltaproteobacteria* also contained genes encoding for cytochrome *aa*_3_-600 oxidase (*qoxABCD*), a heme-copper oxygen reductase that uses ubiquinol or menaquinol in place of cytochrome *c* in the canonical respiration pathway [48]. As shown here, many Hg methylator genomes contained more than one type of terminal oxidase. Such a combination of multiple terminal oxidases has been observed for other microorganisms, facilitating electron transfer to O_2_ under variable redox potentials. In contrast, no genes involved in O_2_ respiration were observed in *hgcA*-carrying Calditrichaeota, Verrucomicrobia, Spirochaetes, and Firmicutes genomes identified from databases. Although only partial operons for some terminal respiratory enzyme complexes were found in some MAGs, possibly resulting from incomplete genome recovery, the complete cytochrome *bd* complex was found encoded by most *Deltaproteobacteria*, suggesting a capability for O_2_ tolerance.

Genes involved in carbohydrate degradation pathways were found in many *hgcA*-carrying MAGs, especially in Marinimicrobia, Verrucomicrobia, and Spirochaetes. These MAGs also represent versatile metabolic capabilities in nitrogen and sulfur cycling. Marinimicrobia MAGs contained complete operons for utilizing nitrogen species in electron transfers, e.g., hydroxylamine oxidoreductase (*hao*), nitrite oxidase (*nxrAB*), nitrate reductase (*narGHI*), and hydrazine dehydrogenase (*hdh*). *Deltaproteobacteria* MAGs presented the potential for some degree of sulfur utilization, for which SAR324 were the only group to possess the gene *ddhA* encoding for a subunit of dimethyl sulfide dehydrogenase. Notably, most *hgcA*-carrying MAGs encoded for sulfide-quinone oxidoreductase (*sqr*), known to oxidize hydrogen sulfide to elemental sulfur for respiration and sulfide detoxification [49]. Nearly all MAGs harbored *mttB* genes encoding for trimethylamine methyltransferase, suggesting a capability for anaerobic one-carbon metabolism. Most MAGs also encoded genes for arsenic reduction (*arsABCDR*), and Cu (*copA*) and Fe (*feoB*) transportation. Furthermore, genes encoding for flagellar proteins were identified in most putative Hg-methylators, enabling these bacteria to reposition optimally within redox gradients to suitable O_2_ and nutrient levels.

Our findings point toward a previously unrecognized marine microbial Hg methylator, and are consistent with previously observed MeHg profiles in marine redoxclines. However, we acknowledge that cultivation experiments with *hgcAB*-bearing Marinimicrobia isolates are ultimately needed both to elucidate their lifestyle and confirm functionality for Hg methylation.

## Materials and methods

### Mercury analysis

Seawater samples for mercury analysis were collected from Saanich Inlet S3 station (48°35.500 N, 123°30.300 W, Fig. 1A) in April 2010. Total and methylmercury (monomethylmercury) determinations were made using USEPA standard methods 1631-E and 1630, respectively [50, 51]. In brief, total Hg was determined on filtered water samples following wet chemical oxidation by BrCl, followed by reduction by NH_2_OH and SnCl_2_, rendering all Hg species as volatile Hg(0). Hg was purged from solution by N_2_ and concentrated on a gold-coated sand cartridge, which was then heated, releasing Hg for re-concentration on a second gold cartridge for final quantification by cold-vapor atomic fluorescence spectrometry (CVAFS; Tekran 2600). Methylated Hg was first separated from the seawater matrix by KCl/H_2_SO_4_/CuSO_4_ extraction and steam distillation. Removal from seawater allowed for derivatization of MeHg into methylethyl-Hg through the use of sodium tetraethylborate, which is volatile and can be purged from solution and pre-concentrated on Tenax. The MeHg derivative was then separated from other Hg forms on a packed gas chromatography column of 15% OV-3 on Chromasorb-W at 110 °C, and then rendered into Hg^0^ through pyrolysis for quantification by CVAFS. Detection limits for total and methylated Hg (3σ of reagent blanks) were 0.5 and 0.1 pM, respectively.

### Multi-omics data description

The multi-omic (metagenomic, metatranscriptomic, and metaproteomic) time-series samples from Saanich Inlet were taken from 2008 to 2013. A total of 84 genome-resolved metagenomic samples (Table S4) were employed in this study, including 78 samples from Saanich Inlet midpoint station “S3” (48°35.500 N, 123°30.300 W), 6 samples from the inlet mouth station “S4” (48°38.310 N, 123°30.007 W) and the inlet end station “S2” (48°33.148 N, 123°31.969 W; Fig. 1A). A set of shotgun metatranscriptomic raw data corresponding to 36 metagenomic samples (Table S4) was used to investigate the transcriptional activity of genes of interest. Protein sequences predicted from the Saanich Inlet metaproteomic dataset [52] were also used to confirm the expression of target proteins in the environment. Sample collection, DNA/ RNA/ protein extraction, and sequencing methods of the datasets were described previously [24]. Briefly, seawater samples spanning six major depths (10, 100, 120, 135, 150, and 200 m) were collected and filtered onto 0.22 μm Sterivex (Millipore) filters. 1.8 ml of RNAlater (Ambion) was added to metatranscriptomic sample filters, and 1.8 ml of sucrose lysis buffer was added to metaproteomic sample filters. Filters were stored at −80 °C until processing. For metagenomic samples, Sterivex filters were thawed on ice and incubated at 37 °C for 1 h with lysozyme (Sigma). Proteinase K (Sigma) and 20% SDS were added subsequently and incubated at 55 °C for 2 h with rotation. Filters were rinsed with sucrose lysis buffer after lysate was removed. Combined lysate was extracted with phenol-chloroform followed by chloroform. The aqueous layer was washed with TE buffer (pH 8.0) for three times and concentrated to 150–400 μl. The metagenomic samples were sequenced at the DOE Joint Genome Institute (JGI) and sequenced on the Illumina HiSeq 2000 platform. For metatranscriptomic samples, total RNA was extracted using the mirVana miRNA Isolation kit (Ambion) modified for Sterivex filters. RNAlater was removed from thawed filters by extrusion and rinsed with Ringer’s solution (Sigma). After incubation at 25 °C for 20 min with rotation, Ringer’s solution was removed by extrusion. Lysozyme was added, following by incubating at 37 °C for 30 min with rotation. Lysate was removed into 15 ml tube and subjected to organic extraction. TURBO DNA-free kit (Thermofisher) was used to remove DNA and the RNeasy MinElute Cleanup kit (Qiagen) was used for purification. Metatranscriptomic shotgun libraries were generated at the JGI on the HiSeq 2000 platform. For metaproteomic samples, Bugbuster (Novagen) was added to thawed filters and incubated at 25 °C for 20–30 min with rotation. Filters were rinsed with 1 ml lysis buffer after removing lysate. Combined lysate was then subject to buffer exchange using Amicon Ultra 10K (Millipore) with 100 mM NH_4_HCO_3_ for three times. Urea was added to a final concentration of 8 M and dithiothreitol added to a final concentration of 5 mM. Samples were incubated at 60 °C for 30 min and diluted ten times with 100 mM NH_4_HCO_3_, following by digesting at 37 °C for 6 h with trypsin. C18 solid phase extraction and strong cation exchange were carried out subsequently. Tandem mass spectrometry (MS/MS) was used to sequence the protein samples, and MS-GFDB [53] was used to identify peptides based on the matched Saanich Inlet metagenomic sequences.

### Metagenomic assembly and binning

The raw Illumina reads from metagenomic samples (2 × 150 bp paired-end, median 13.75 Gbp per sample) were first filtered and trimmed by Trimmomatic v3.6 [54]. Samples from the same station and time point were co-assembled with MEGAHIT v1.2.8. MAGs were recovered using MetaBAT v2.14 [55] and MaxBin v2.2.7 [56], which were then merged and refined with MetaWRAP v1.2 [57]. In order to further improve the quality of the MAGs, metagenomic reads were remapped to each MAG and then reassembled by SPAdes [58] in careful mode. The qualities of derived MAGs were examined using CheckM [59]. 16S rRNA genes were predicted by RNAmmer [60] and then used to assign taxonomic classifications of the MAGs. For those MAGs that lacked 16S rRNA genes, GTDB-Tk v0.3.2 [61] was used to predict taxonomies according to genome contents. Genome annotation was conducted with Prokka v1.14 [62]. KEGG pathways of MAGs of interest were predicted with BlastKOALA (http://www.kegg.jp/blastkoala/), and completeness of the pathways was estimated by KEGG-Decoder [63] according to the KEGG Pathway Database (https://www.genome.jp/kegg/pathway.html).

### Relative abundance calculation

CD-HIT v4.6 [64] was used to choose representative *hgcA* sequences from all the MAGs under a 99% sequence identity threshold. Paired-end reads were remapped to these *hgcA* sequences with BWA-MEM algorithm [65]. BBMap (http://sourceforge.net/projects/bbmap/) was used to calculate the average coverage. MicrobeCensus [66] was used to estimate the genome equivalent of every sample. The relative abundance of every representative *hgcA* in every sample was calculated by the formula “*hgcA* coverage / genome equivalent”; relative abundance of *hgcA* genes was assumed to represent that of corresponding MAGs, since all reported methylators only carry one copy of *hgcA* gene in the genome. To evaluate and compare expression levels of genes of interest, RPKM values were calculated for each gene, normalized for both gene length and metatranscriptomic sequencing depth. RPKM values for housekeeping genes *rpoB* and *gyrB* were also calculated to normalize the expression of *hgcA*, with the ratio of *hgcA*/*rpoB* RPKM and *hgcA*/*gyrB* RPKM representing the expression of *hgcA* in individual microorganism regardless of the abundance variation of the total *hgcA*-carrying microorganisms.

### Search for *hgcAB* genes in MAGs

An in-house database of experimentally validated functional HgcAB sequences (see Table S5) was used to search for HgcAB encoding genes in MAGs by BLASTp, and BLAST results were further confirmed by examining the existence of conserved motifs ([N(V/I)WCA(A/G)(A/G)K] in HgcA and [CX2CX2CX3C] in HgcB), respectively. All the derived MAGs with *hgcAB* were then proceeded by the script esomWrapper.pl [67] to determine tetra-nucleotide frequencies signatures, followed by generating ESOM with Databionic emergent self-organizing map tools (http://databionic-esom.sourceforge.net/). The presence of *hgcAB* fragments in the MAGs were confirmed by checking manually in the ESOM.

### Phylogenetic analysis

HgcA sequences from the two most recent papers [14, 15] were retrieved and combined with the HgcA sequences predicted from genome taxonomy database (GTDB, release 89, https://gtdb.ecogenomic.org/) to establish an HgcA database (see Table S6 for accession numbers), after the redundant sequences were eliminated by CD-HIT [64] at 50% cutoff. These sequences were aligned with the putative HgcA sequences from Saanich Inlet by MAFFT v7 with the high-sensitivity (L-INS-i) algorithm [68]. The alignment was trimmed by trimal v1.2 [69], followed by the maximum likelihood (ML) tree was reconstructed by IQ-TREE v1.6 [70] under the LG + F + R9 protein substitution model chosen according to BIC. Publicly available Marinimicrobia 16S rRNA genes (see Table S7) were retrieved from the SILVA database (release 138) [36] and aligned with the 16S rRNA genes of *hgcA*-carrying Marinimicrobia to build an ML tree using IQ-TREE v1.6. The ML tree of all the Marinimicrobia-affiliated MAGs found in this study, as well as in public databases (see Table S8), was built by PhyloPhlAn2 [71] using 400 universal proteins without duplication.

### Searching SRA database

To investigate the distribution of Marinimicrobia*-hgcA*, the recovered Marinimicrobia*-hgcA* gene nucleotide sequence was first employed as a query to be searched in the NCBI SRA database using the SRA search tool [72], which is able to map ~1% reads of more than 100,000 public whole shotgun metagenomic samples to the query sequences using Bowtie2 [73]. Samples that contained reads associated with the Marinimicrobia*-hgcA* gene were then checked by downloading the corresponding read set from the SRA database and mapping these reads to the *hgcA* nucleotide sequence.

### Protein homology modeling

The three-dimensional structures of the putative HgcA and HgcB sequences found in this study were built and refined using Schrödinger suit v2012 (LLC, New York, USA). The transmembrane regions were predicted by CCTOP [74]. HgcAB sequences were searched against the protein data bank (PDB) using BLASTp with a word size of three. The structure of the corrinoid iron-sulfur protein (CFeSP) AcsC from *Carboxydothermus hydrogenoformans* (PDB ID: 2H9A; 1.9 Å; 28% identity and 55% similarity to Marinimicrobia-HgcA) and the CFeSP AcsC from *Moorella thermoacetica* (PDB ID: 4DJD; 2.38 Å; 31% identity and 59% similarity to Marinimicrobia-HgcA) were identified as appropriate templates for homology modeling of the HgcA N-terminus globular structured domain, according to their similarities and the resolution at which they were solved. The structure of the transmembrane bacteriorhodopsin Bop (PDB ID:2ZFE; 2.5 Å) was used to model the transmembrane region of HgcA, due to their comparable secondary structures. The homology model of HgcB proteins was built using the experimental structures of iron hydrogenase HydA from *D. desulfuricans* (PDB ID: 1HFE; 1.6 Å; 36% identity and 53% similarity to Marinimicrobia-HgcB) and the flavin-based caffeyl-CoA reductase CarE (PDB ID: 6FAH_A; 3.133 Å; 39% identity and 45% similarity to Marinimicrobia-HgcB) in a similar procedure. The stereochemical quality and accuracy of reconstructed homology models were assessed and compared to each other by generating a Ramachandran plot using the Rampage server [75] and a best model was selected. Hydrogen bonding between HgcA protein and the ligand cobalamin were predicted by Arpeggio [76], PLIP [77], Chimera [78], and NGL Viewer [79], and consistent hydrogen bonding among the four programs was considered to be robust. Homology models for HgcAB from the Hg-methylating model strain *D. desulfuricans* ND132 were also built by the methods described above.

## Supplementary information

Table S1

Table S2

Table S3

Table S4

Table S5

Table S6

Table S7

Table S8

Supplemental Figures

## Data Availability

Data from this project have been deposited at DDBJ/ENA/GenBank under Project ID PRJNA630981. The 2088 MAGs generated by this study are available with accession numbers JABGOO000000000 to JABJQV000000000.

## Code availability

Customized scripts for conducting metagenomic and metatranscriptomic analyses are available from GitHub (https://github.com/SilentGene/MultiOmicAnalysis).
