# Supplementary material for: Mercury methylation by metabolically versatile and cosmopolitan marine bacteria
Source: ISME J. 2021 Jan 27;15(6):1810–25. doi: 10.1038/s41396-020-00889-4 (PMC8163782; doi:10.1038/s41396-020-00889-4)
Supplement: Supplementary file 9 — Supplemental Figures [file 41396_2020_889_MOESM9_ESM.pdf]

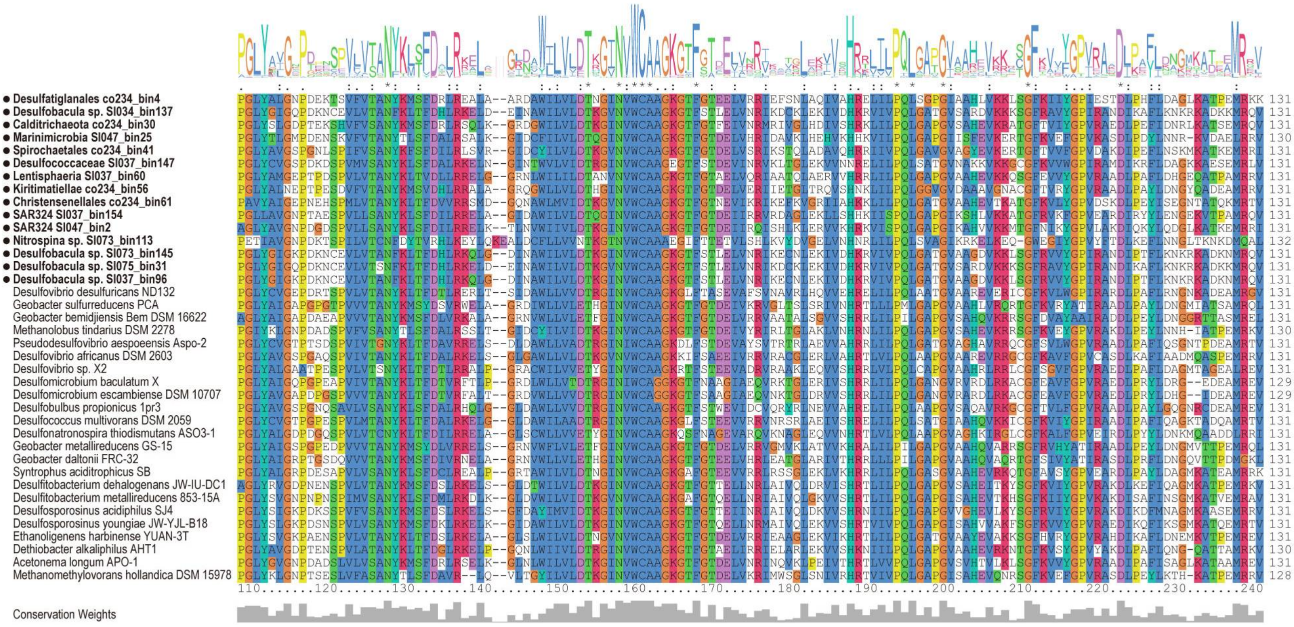

Figure S1. Multiple *hgcA* sequence alignment. Alignment shows 15 partial representative *hgcA* sequences found in this study (highlighted in bold and with black dots) and other experimentally confirmed *hgcA* sequences. Sequence logo above the alignment shows the occurrence of each amino acid in each position. Conservation of each sequence position is shown below the alignment.

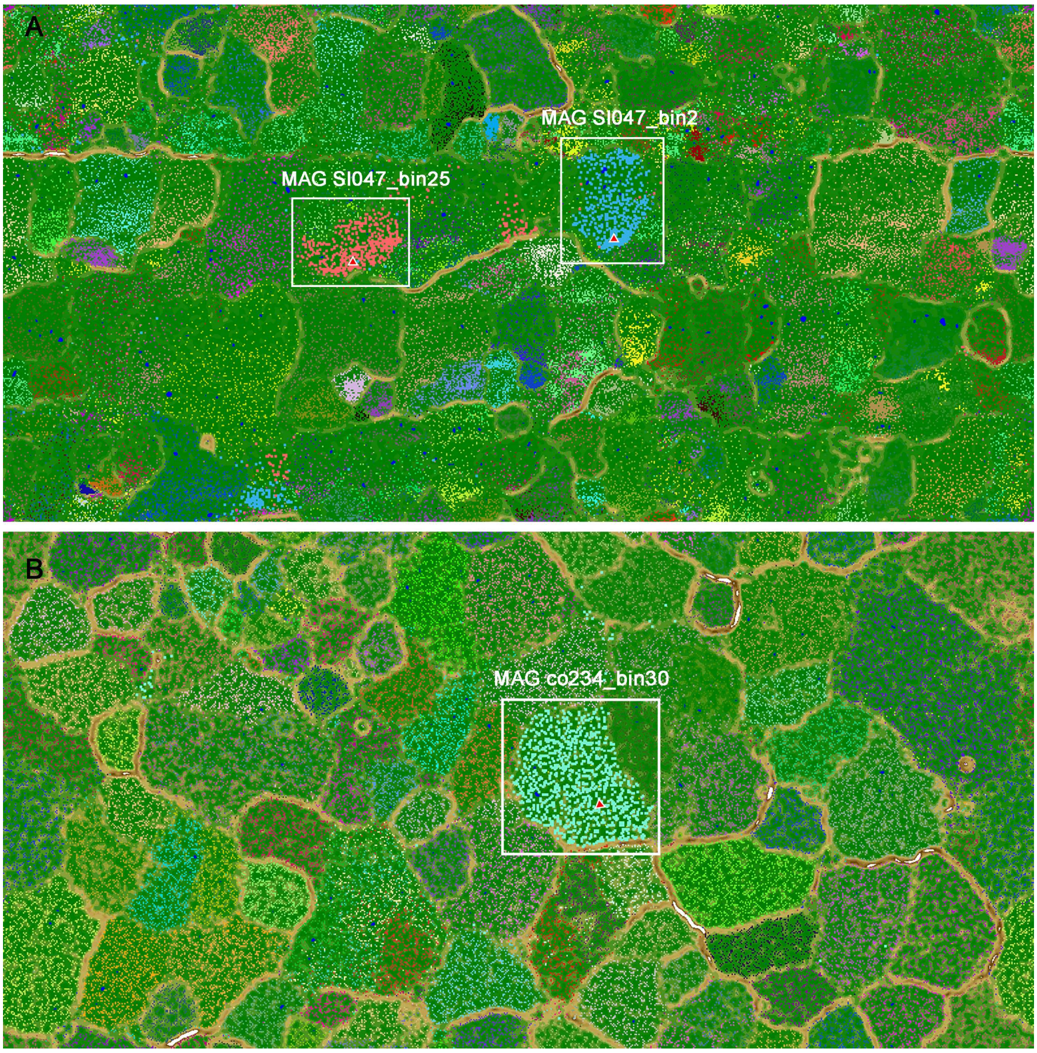

Figure S2. Emergent self-organising maps (ESOM). ESOMs show three representative novel *hgcA*-carrying MAGs recovered from Saanich Inlet metagenomic datasets. Dots with different colors represent DNA fragments from different MAGs. (A) ESOM map built according to 131 MAGs recovered together with *Marinimicrobia* MAG SI047_bin25 and SAR324 MAG SI047_bin2, with the two white squares indicating DNA fragments that belonged to each of two targeted MAGs, respectively. Red triangles with white edges indicate fragments containing *hgcA* genes binned with other fragments. (B) ESOM map built according to 97 MAGs recovered together with *Calditrichaeota* MAG co234_bin30, with white square indicating DNA fragments that belonged to MAG co234_bin30. Red triangle with white edge indicates the fragment containing the *hgcA* gene.

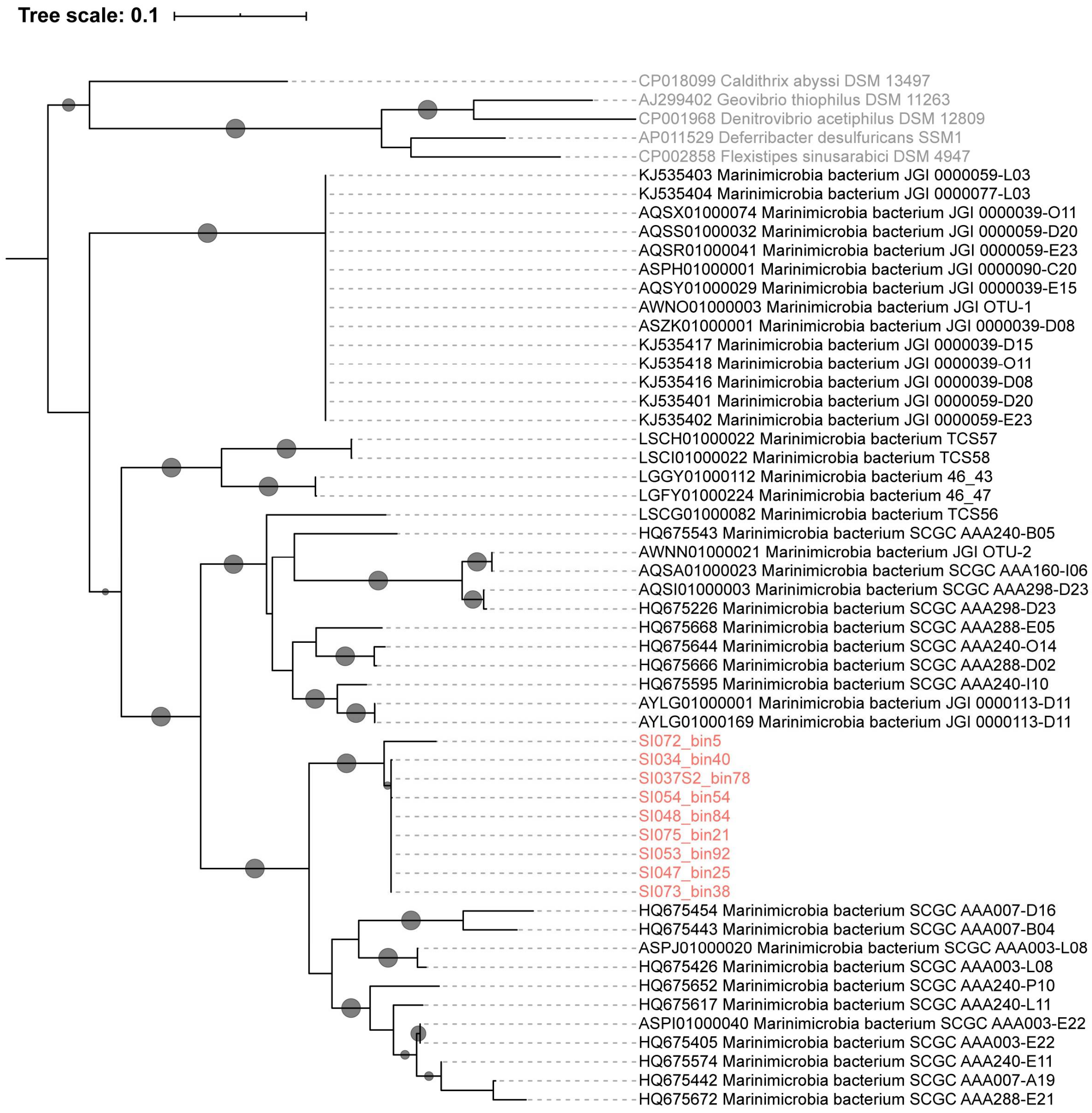

Figure S3. Maximum-likelihood phylogenetic tree of 16S rRNA genes. Tree was built from *hgcA*-carrying *Marinimicrobia* (red) and reference *Marinimicrobia* (black) from SILVA database. Branch support analysis was evaluated by 1000 ultrafast bootstrap replicates, and values >90% are shown by black dots at nodes.

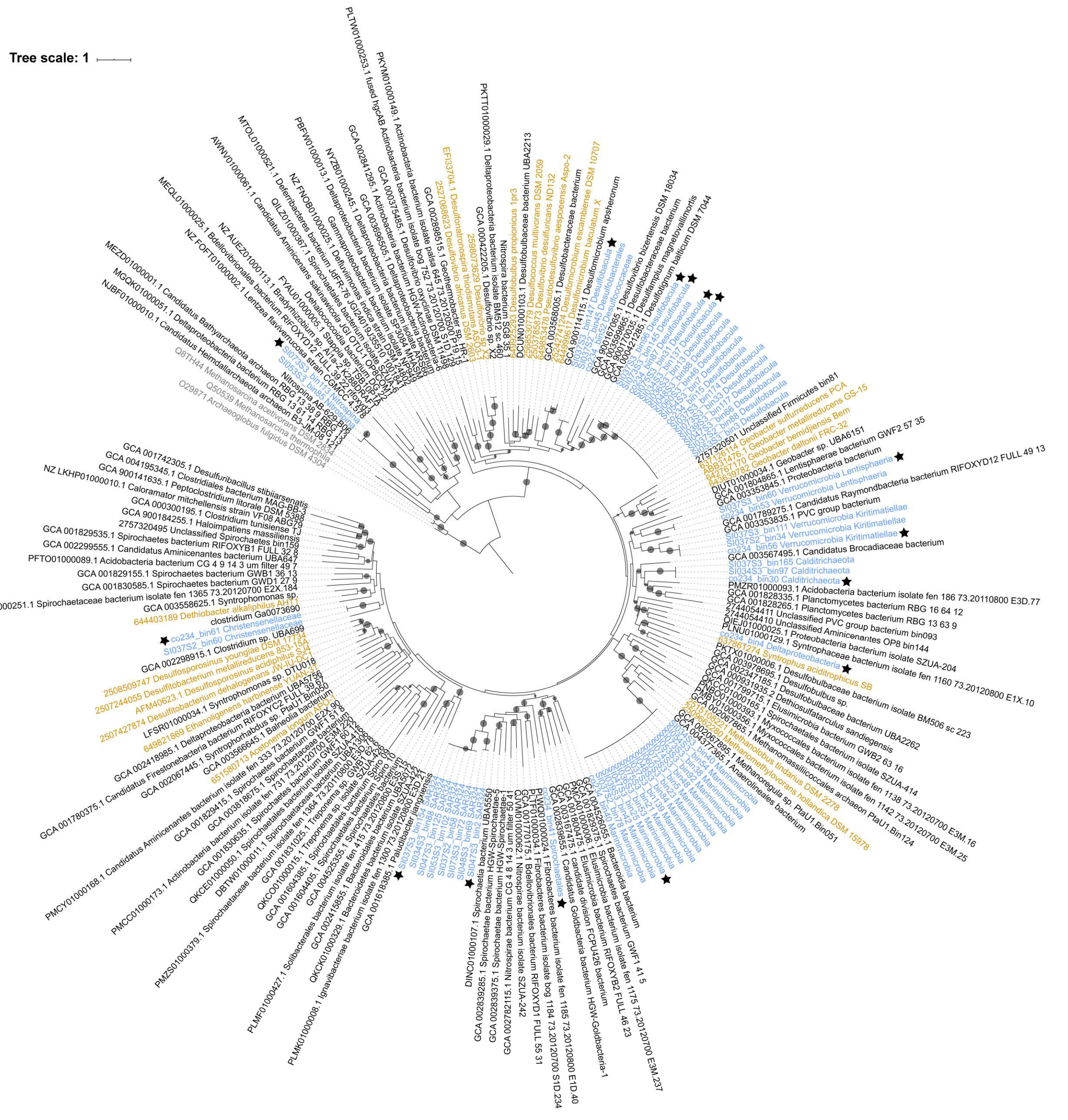

Figure S4. Maximum-likelihood phylogenetic tree of HgcA amino acid sequences (1,000 ultrafast bootstrap replicates; values >90% are shown by black dots at the nodes). This is a detailed tree of Figure 2. HgcA sequences recovered in this study are highlighted in blue. HgcA sequences retrieved from public databases with corresponding genome IDs are shown in black. HgcA paralogues from non-methylating bacteria are used as outgroups and are shown in grey. Experimentally confirmed HgcA from previous studies are shown in brown. The 15 representative HgcA sequences used in this study are indicated by stars. Taxonomic classifications of the *hgcA*-carrying genomes are labeled in the outer circle by different colors. Scale bar indicates substitutions per site.

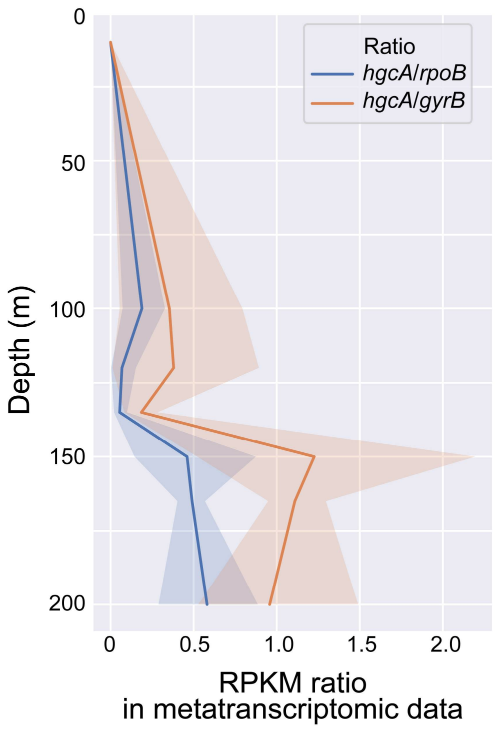

Figure S5. Abundance of *hgcA* transcripts in Saanich Inlet datasets normalized by *rpoB* and *gyrB*. Line plotted in deep blue and deep orange depicts the ratio of *hgcA/rpoB* and *hgcA/gyrB* with depth, respectively; light blue and light orange areas depict 95% confidence intervals.

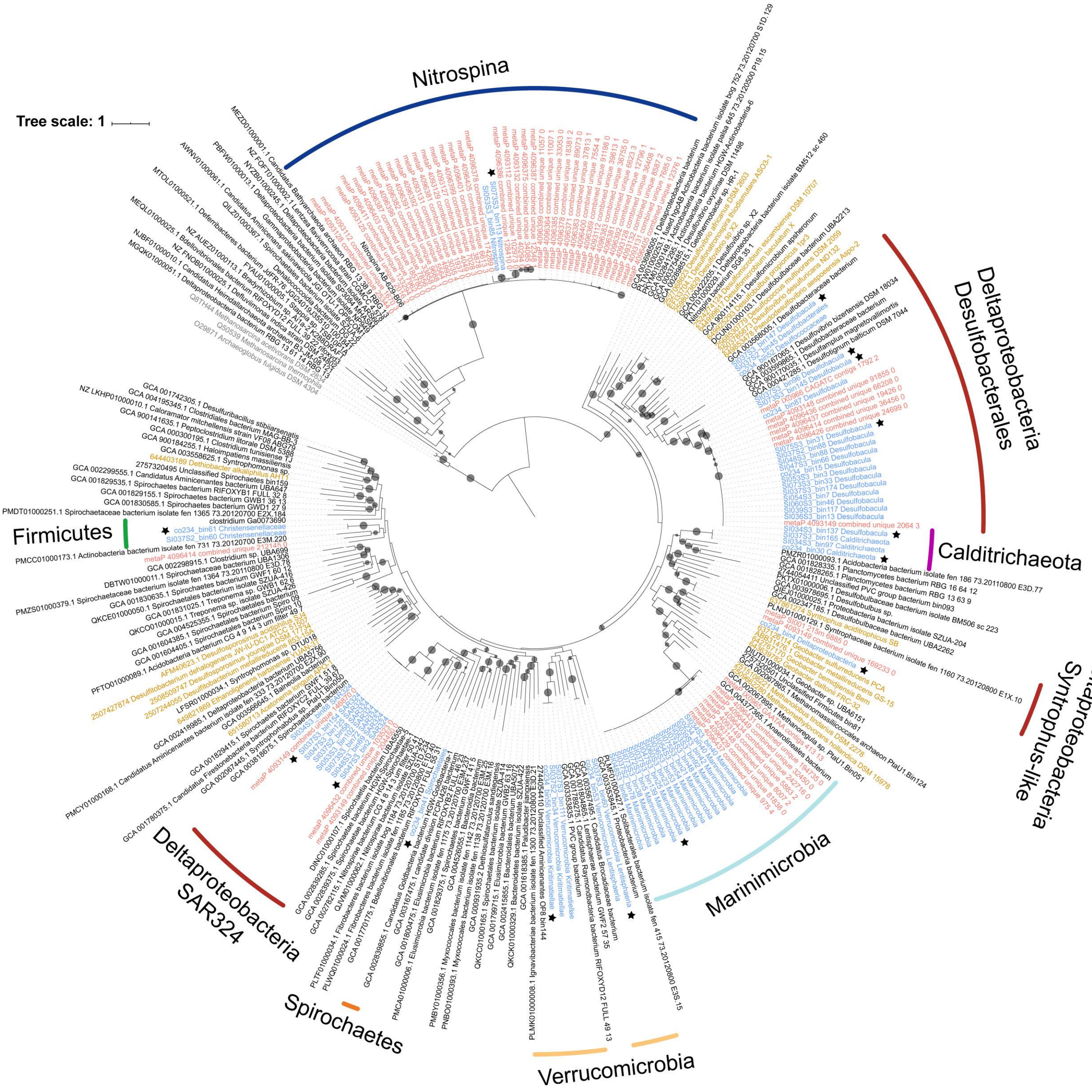

Figure S6. Maximum-likelihood phylogenetic tree of HgcA sequences. Tree was constructed from metagenomic (blue) and metaproteomic (red) datasets from Saanich Inlet (1,000 ultrafast bootstrap replicates; values >90% are shown by black dots at nodes). Predicted HgcA sequences from public databases and corresponding gene IDs are shown in black as references. Experimentally confirmed HgcA from previous studies are shown in brown. HgcA paralogues from non-methylating bacteria (shown in grey) were used as outgroups. The 15 representative HgcA sequences used in this study are indicated by stars. Taxonomic classifications of Hg methylators are labeled in the outer circle with different colors. Scale bar indicates substitutions per site.

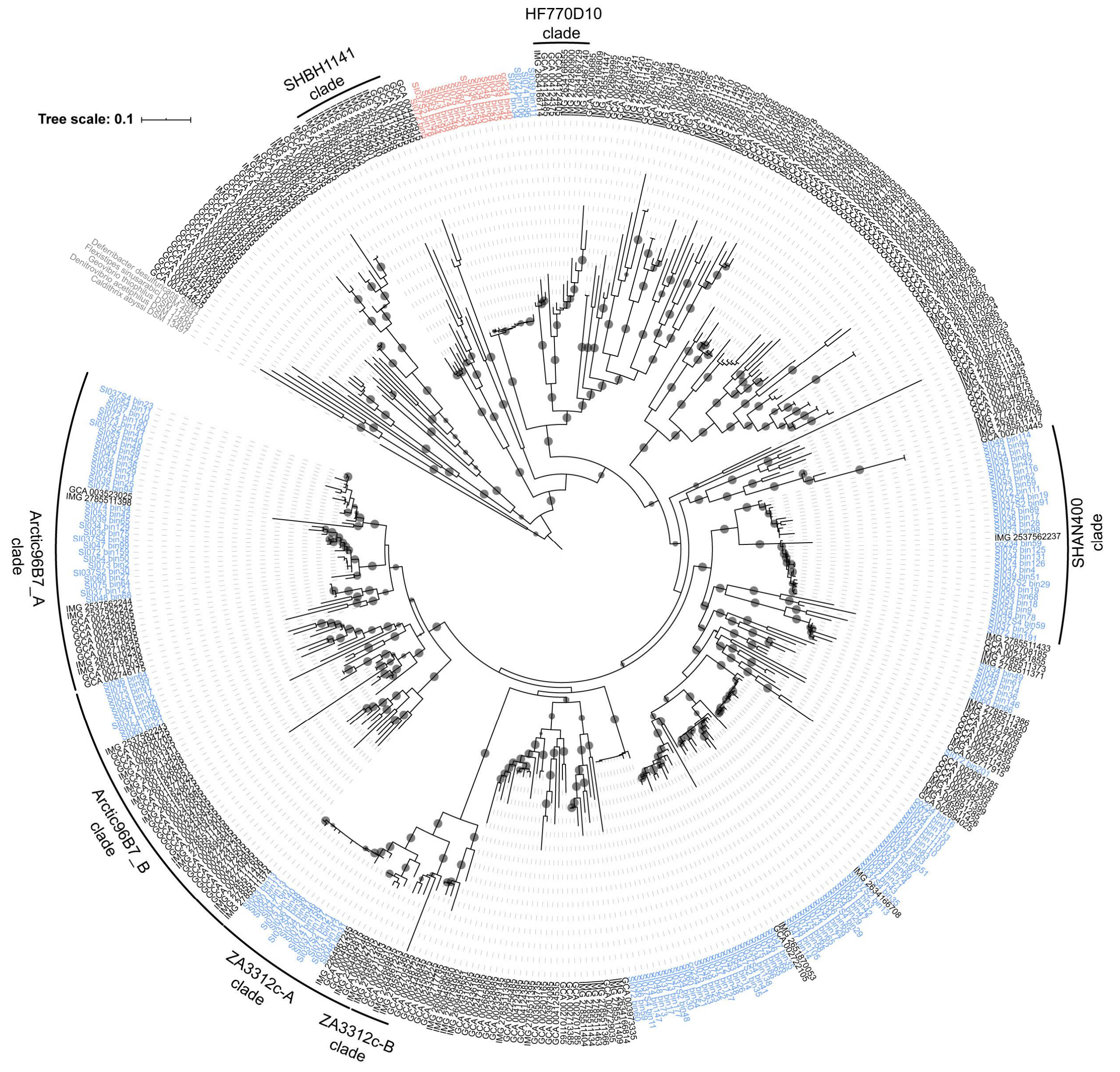

Figure S7. Phylogenetic tree of the phylum *Marinimicrobia*. Tree is based on concatenated housekeeping genes (1,000 ultrafast bootstrap replicates; values >90% shown by black dots at nodes). A total of 424 *Marinimicrobia* genomes were used to construct the tree. The 15 *hgcA*-carrying *Marinimicrobia* MAGs are shown in red, while other *Marinimicrobia* MAGs recovered in this study are shown in blue. *Marinimicrobia* genomes from public databases (GenBank and IMG) are shown in black, with associated accession numbers shown. Five genomes from other related phyla were used for an outgroup, and are shown in grey. *Marinimicrobia* clades as defined by Hawley et al (2017) are labeled in the outer circle. Scale bar indicates substitutions per site.

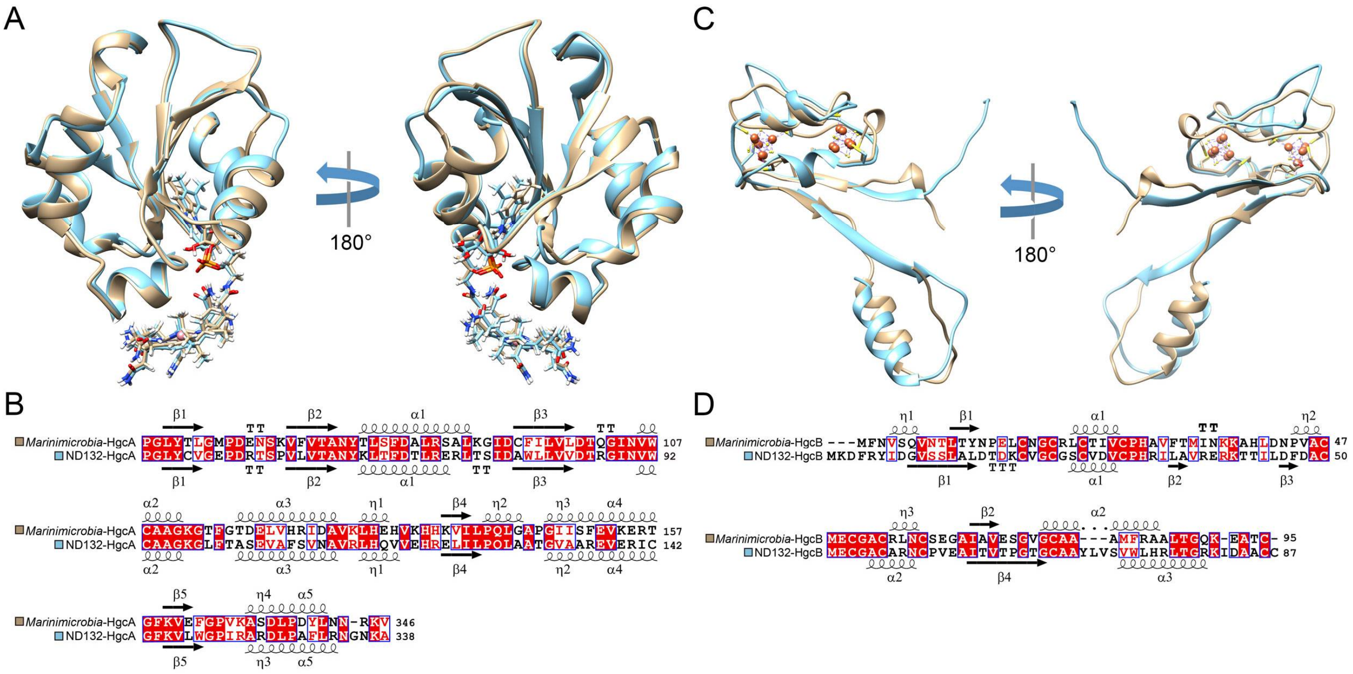

Figure S8. Structure and sequence comparison between HgcAB from *Marinimicrobia* and *Desulfovibrio desulfuricans* ND132. (A) Superposition of *Marinimicrobia*-HgcA (grey) and ND132-HgcA (blue) homology models. (B) Sequence alignment of *Marinimicrobia*-HgcA and ND132-HgcA amino acid sequences; secondary structure elements are indicated. A red box over a white character highlights a strict identity, a red character indicates a similarity in a group, and a blue frame indicates a similarity across groups. (C) Superposition of *Marinimicrobia*-HgcB (grey) and ND132-HgcB (blue) homology models. (D) Sequence alignment of *Marinimicrobia*-HgcB and ND132-HgcB amino acid sequences; secondary structure elements are indicated. A red box over a white character highlights a strict identity, a red character indicates a similarity in a group, and a blue frame indicates a similarity across groups.

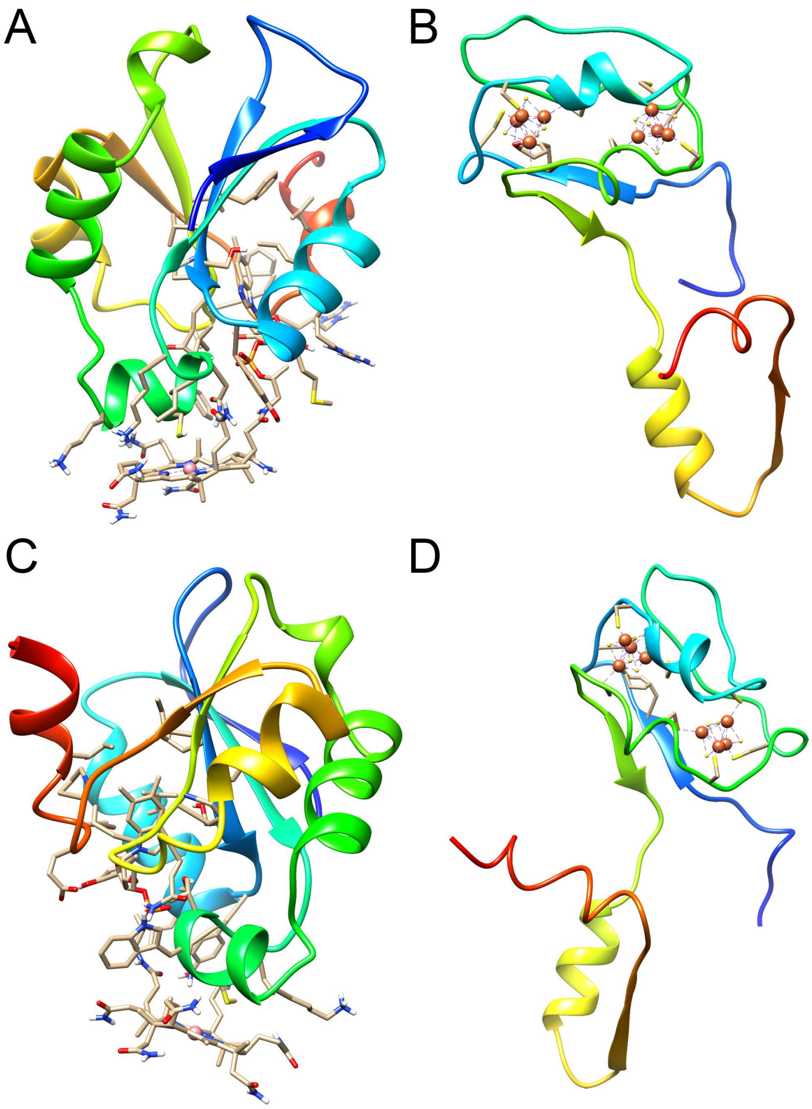

Figure S9. Homology models of HgcA and HgcB proteins. (A) *Calditrichaeota* HgcA model (globular domain) complexed with cobalamin. (B) *Calditrichaeota* HgcB model complexed with two [4Fe4S] ligands. (C) SAR324 HgcA model (globular domain) complexed with cobalamin. (D) SAR324 HgcB model complexed with two [4Fe4S] clusters.
